# Evaluation of a Simplified Technique for the Excision of Non-palpable Breast Cancer

**DOI:** 10.7759/cureus.103577

**Published:** 2026-02-14

**Authors:** Aya M Alamrawy, Mohamed Alatrash, Lamiaa Adel, Emad Khallaf

**Affiliations:** 1 General Surgery, Cairo University, Cairo, EGY; 2 Breast Surgery, Guy's and St Thomas' NHS Foundation Trust, London, GBR; 3 General Surgery, Maidstone and Tunbridge Wells NHS Trust, Tunbridge Wells, GBR; 4 General and Colorectal Surgery, Cairo University, Giza, EGY; 5 Radiology, Cairo University, Giza, EGY; 6 Surgery, Cairo University, Giza, EGY

**Keywords:** non-palpable breast cancer, orthogonal mapping, re-excision rate, surgical resection with an adequate margin, wire-guided localization

## Abstract

Breast cancer is the most common malignancy and the leading cause of cancer-related mortality among women, with early detection through screening programs significantly improving survival rates and reducing mortality. Accurate localization and excision of early-stage non-palpable lesions minimize the need for re-excision, prevent unnecessary removal of breast tissue, and improve cosmetic outcomes. This study aimed to optimize the wire localization technique for the excision of non-palpable breast cancer. A total of 46 female patients referred to the breast unit at Kasr Al-Ainy, Cairo University, underwent wire localization excision, with wire insertion performed one day before surgery. Modifications to the localization process included optimized timing of wire insertion, enhanced radiology reporting with diagrams indicating wire distance, direction (clockwise orientation), and orthogonal mapping, as well as placement of a skin mark over the lesion site. In some cases, aqueous carbon suspension was used to improve intraoperative visualization. The incision was made near the skin site corresponding to the lesion, flaps were raised, and the wire was retracted within the wound, followed by dissection along its path with a cuff of normal tissue to ensure adequate margins. The re-excision rate was minimal and was all due to ductal carcinoma in situ at the margins. This study demonstrated that the simplified excision technique was safe, reproducible, and effective for the management of non-palpable breast cancers, offering a reliable and reproducible orthogonal map, which was found to help with minimizing the re-excision rate without any additional resources.

## Introduction

Breast cancer is the most prevalent cancer among women globally, and early detection plays a vital role in reducing mortality rates. With the advent of screening, a rising percentage of breast excisions are for non-palpable lesions [1]. Advancements in screening programs have led to the earlier detection of breast cancers, many of which present as non-palpable lesions identifiable only through imaging. These clinically occult lesions, whether benign or malignant, pose distinct challenges for surgical management, requiring precise localization to ensure effective and tissue-conserving excision [2].

Over the past few decades, several localization techniques have been developed to guide the surgical excision of non-palpable breast lesions. Wire-guided localization (WGL), first introduced in 1966, remains a widely used and reliable method due to its simplicity and cost-effectiveness. However, it presents notable challenges, including logistical constraints, patient discomfort, and the risk of wire migration [3]. Intraoperative ultrasound (IOUS) offers the advantage of real-time lesion visualization and has demonstrated higher rates of complete excision with negative margins, though its application is limited to lesions visible on ultrasound imaging. The use of breast biopsy markers has enhanced localization accuracy without the need for wires, but marker visibility on imaging continues to pose limitations despite design improvements. Radioactive techniques such as radio-guided occult lesion localization (ROLL) and radioactive seed localization (RSL) allow for greater scheduling flexibility and improved patient comfort, but they necessitate specific equipment and multidisciplinary coordination due to radiation safety concerns [3].

In recent years, a variety of wireless non-radioactive techniques have emerged including magnetic seed localization, radar-based systems, and radiofrequency tagging which provide more patient-friendly options and adaptable scheduling. While these innovations have gained popularity for their safety and convenience, their higher cost and the requirement for specialized training remain barriers to widespread adoption. Nevertheless, WGL remains widely used in current practice due to its cost-effectiveness. At our institution, WGL continues to be the gold standard due to its cost-effectiveness. While WGL has clear benefits in terms of cost, efficacy, and a trained workforce, it also carries several weaknesses, including logistical challenges (requiring wire placement on the day of surgery) and the risk of wire displacement. Despite widespread WGL use, a majority of breast surgeons have expressed a preference to switch to alternative localization techniques [4].

Reported re-excision (re-operation) rates after initial breast-conserving surgery using WGL vary widely, ranging from around 10% up to 40% in different series [5]. This study aimed to refine the conventional WGL technique for excising non-palpable breast cancers, with the goal of reducing positive margins and the need for re-excision. We implemented several modifications to the standard WGL workflow to achieve this aim. Specifically, wire placement was performed one day prior to the surgery, surgeons were provided with detailed orthogonal map diagrams of the wire and lesion position, and adjunct intraoperative measures were utilized in select cases (such as marking the skin over the lesion site and circumferentially injecting a carbon suspension around the lesion) to aid localization. We then evaluated the outcomes of this modified approach in a prospective series of 46 patients, examining localization success, margin status, and re-excision rates after the initial surgery.

## Materials and methods

This prospective study was conducted at the Breast Unit of Kasr Al-Ainy School of Medicine, Cairo University, Egypt, between April 2019 and April 2020. Informed consent was obtained from all participants before their inclusion in the study. The study involved 46 female patients who were referred following breast cancer screening. Notably, all participants were asymptomatic and had no prior breast-related complaints before being recalled for further evaluation. The inclusion criteria were female patients with image-detected, non-palpable breast cancer, while patients diagnosed with palpable breast cancer were excluded.

Preoperative wire localization

**Figure 1:**
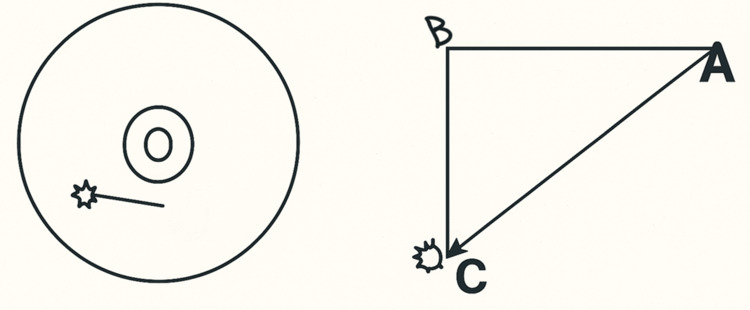
Orthogonal map diagram for ultrasound-guided wire localization The orthogonal map shows wire orientation toward the 7 o’clock position. A marks the wire entry point, B marks the skin point directly above the lesion, and C marks the lesion (hook wire tip). Distances measured are: A–C = 3.19 cm, C–B = 1.92 cm, and A–B = 2.62 cm. Image credit: Created by the authors using Microsoft PowerPoint (Microsoft Corp., Redmond, WA, USA). Some elements were difficult to replicate digitally alone, so hand-drawn sketches were incorporated and then digitized to maintain clarity and educational value.

**Figure 2:**
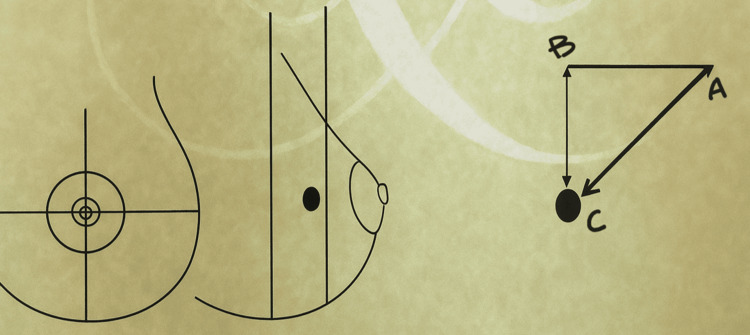
Another example of an orthogonal map The orthogonal map shows wire orientation toward the 3 o'clock position. A marks the wire entry point, B marks the skin point directly above the lesion, and C marks the lesion (hook wire tip). Distances measured are: A–C = 7 cm, C–B = 2.5 cm, and A–B = 2 cm. Image credit: Created by the authors using Microsoft PowerPoint (Microsoft Corp., Redmond, WA, USA). Some elements were difficult to replicate digitally alone, so hand-drawn sketches were incorporated and then digitized to maintain clarity and educational value.

Wire localization was performed one day before surgery at the breast imaging unit of Kasr Al-Ainy (or on the morning of surgery for patients later on the list). Prior to the procedure, all previous imaging studies were thoroughly reviewed, followed by ultrasound-guided wire insertion. To enhance surgical precision, modifications were made to the reporting methods and the description of wire positioning relative to the breast cancer lesion. Additionally, a simplified orthogonal map diagram was included in all wire localization reports to clarify wire placement (Figures 1, 2).

**Figure 3:**
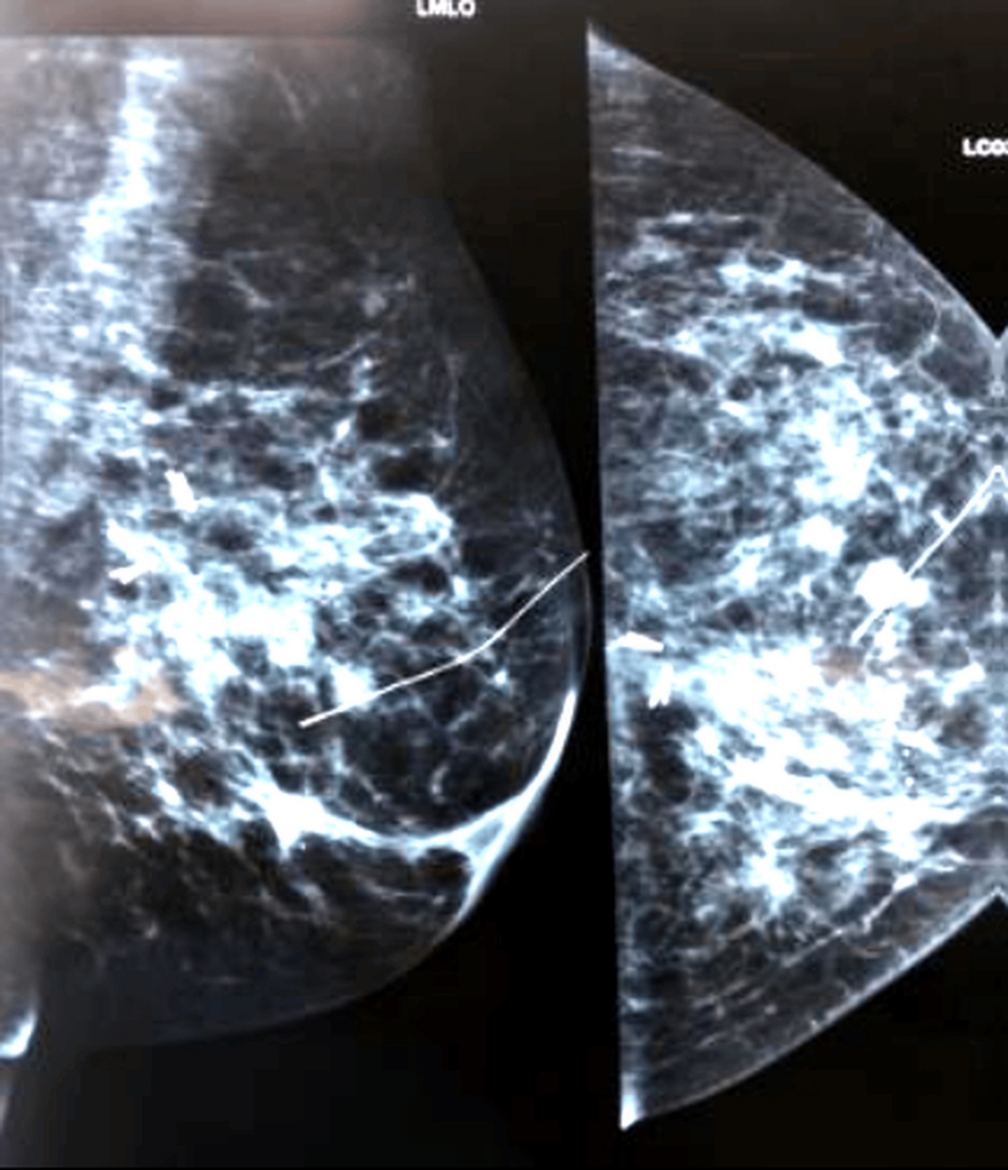
Post-ultrasound-guided wire insertion mammogram The wire is transfixing the lesion.

A post-localization mammogram confirmed that the wire traversed the lesion (Figure 3).

The diagram includes key details such as the direction of the wire in a clock fashion (with the center of the clock being the wire entry point). It also shows the distance between the lesion and the skin surface and the distance between the hook and the wire entry site. This orthogonal mapping system was a valuable tool for surgical orientation, assisting in the precise planning of the incision site to ensure optimal access to the lesion.

**Figure 4:**
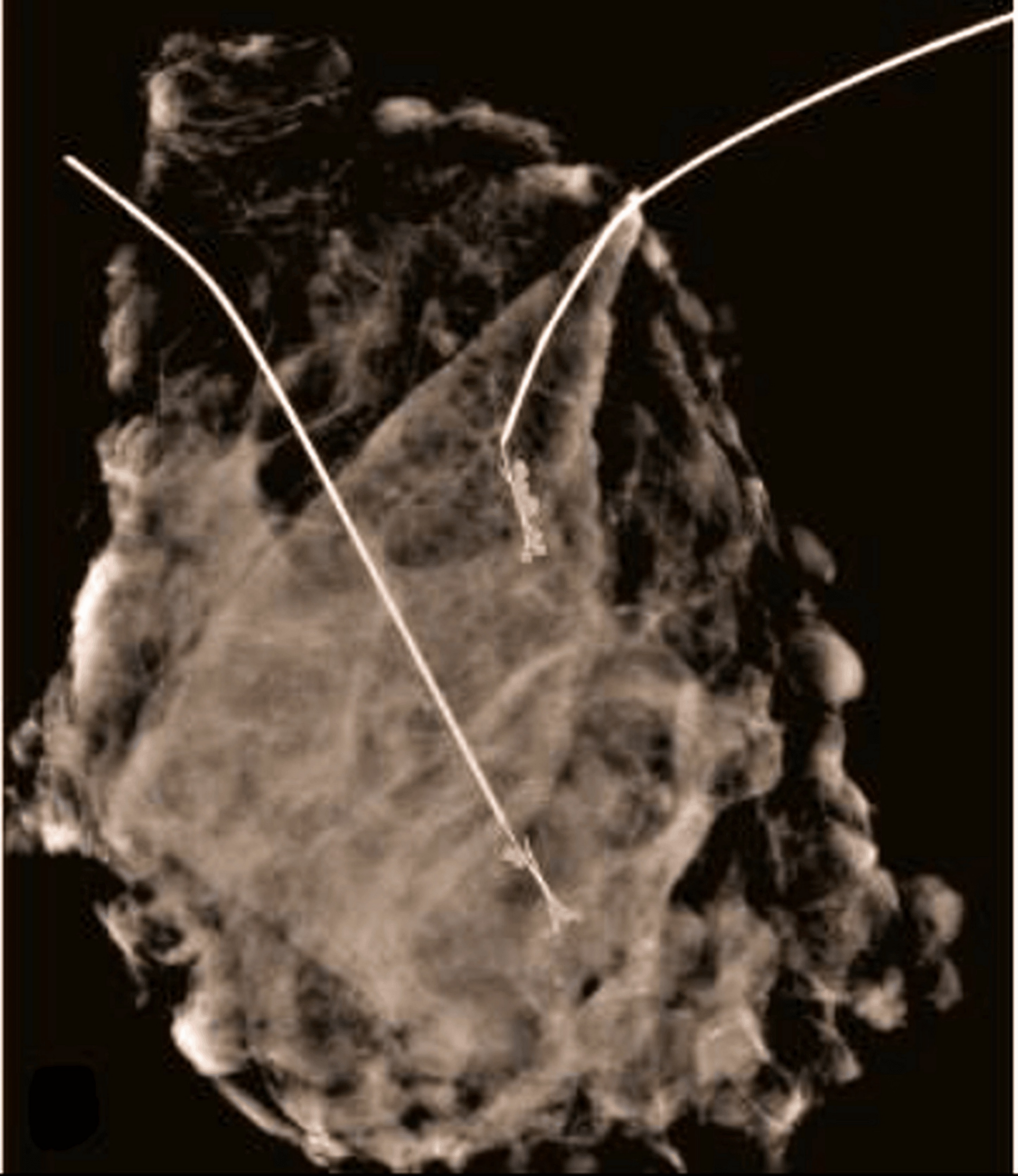
Bracketing wire insertion

**Figure 5:**
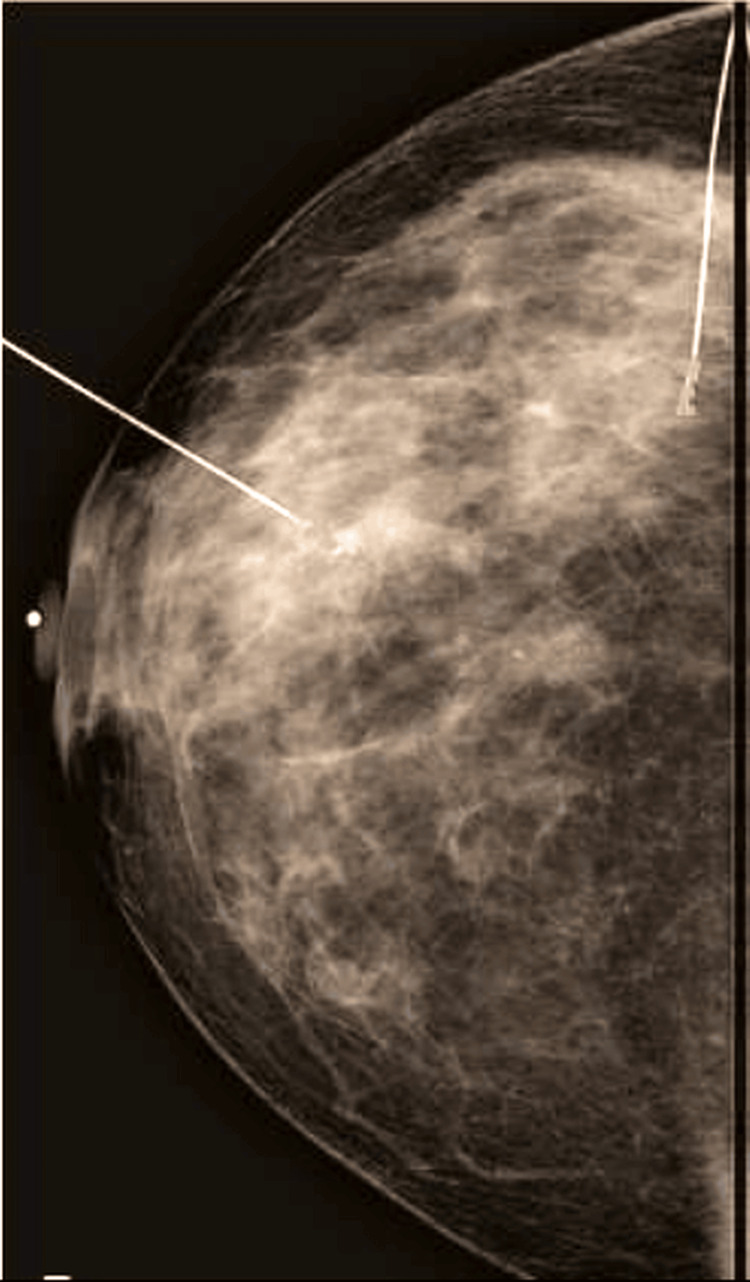
Another example of bracketing wire insertion

For cases with a large area of segmental calcifications, multifocal lesions, or architectural distortion, bracketing wires were used to mark the edges of the lesion and to guide breast-conserving surgery (with or without oncoplastic techniques) as shown in Figures 4, 5.

The excision of an ultrasound-guided wire differed from that of a mammography-guided wire in its insertion technique. Ultrasound-guided wires are typically placed at a distant site, which does not directly correspond to the lesion, whereas mammography-guided wires are inserted perpendicular to the lesion. When planning the incision for an ultrasound-guided wire-localized excision, the incision should be based on the site of the breast lesion rather than the wire entry point, using a common oncoplastic approach (e.g., peri-areolar, inframammary, or curvilinear incision) when feasible. After raising the flaps, the wire was carefully retracted within the wound, and the dissection proceeded along its path, keeping in mind the location of the hook in relation to the lesion and the size of the lesion, in order to ensure an adequate safety margin while avoiding excessive resection of the normal tissue. Routine specimen radiography was performed to confirm that the entire wire and lesion were excised with a clear margin. Margin shave biopsies were routinely taken for intraoperative frozen section analysis. Titanium clips were placed to mark the operative bed prior to closure of the breast parenchyma for guidance during radiotherapy.

Surgical procedure (detailed steps)

**Figure 6:**
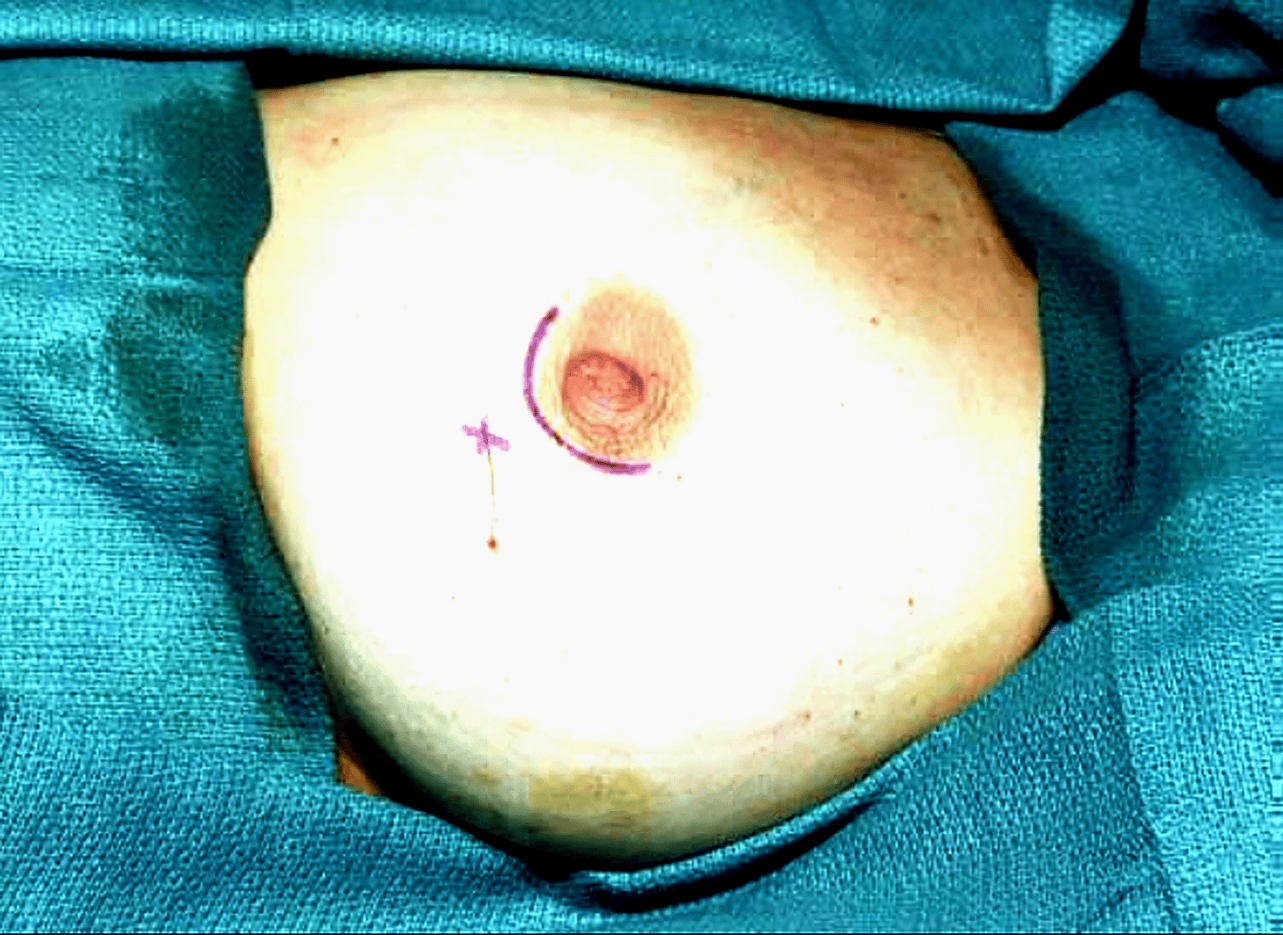
Skin incision was planned directly over the target lesion rather than at the wire’s entry site on the skin

The skin incision was planned at the site of the lesion rather than at the wire entry point whenever feasible, as shown in Figure 6.

**Figure 7:**
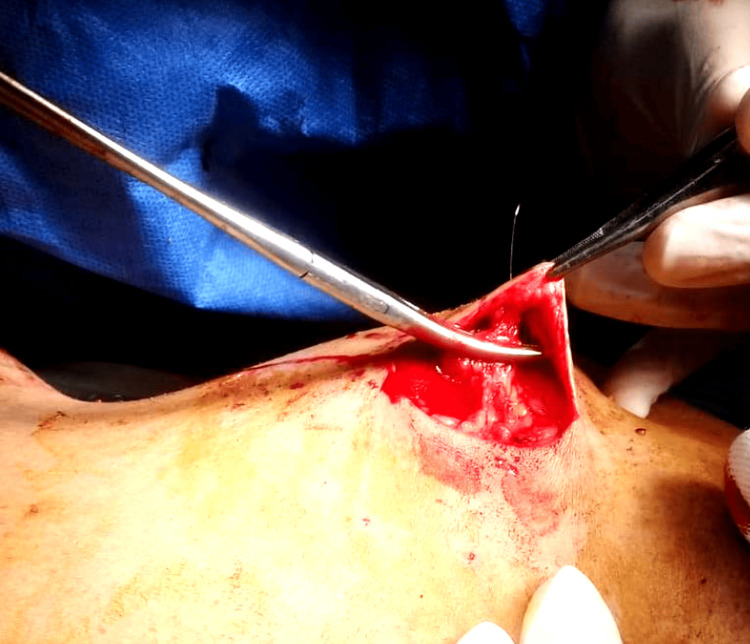
A short skin flap was raised toward the wire’s entry site to facilitate retrieval of the wire through the incision

After making the incision, the cut was deepened through the subcutaneous fat. A short flap was then raised toward the wire entry site to facilitate delivery of the wire into the wound, as shown in Figure 7.

**Figure 8:**
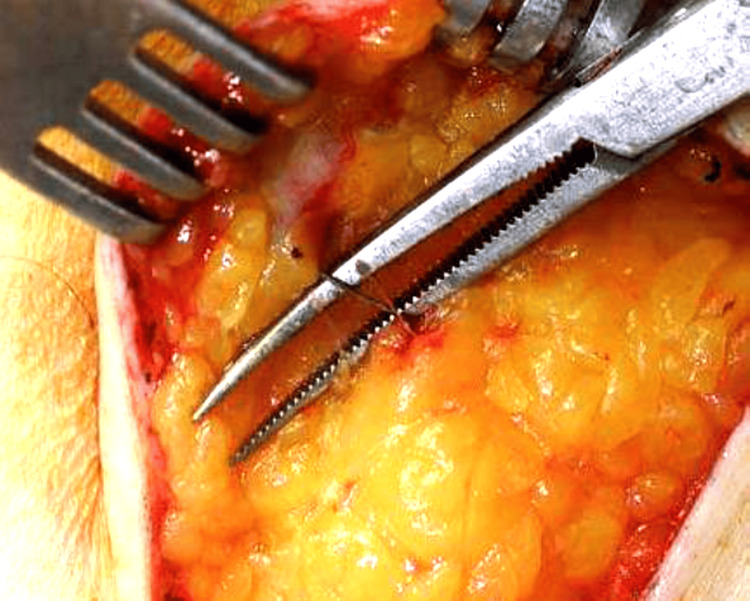
Gradual delivery of the localization wire through the incision

**Figure 9:**
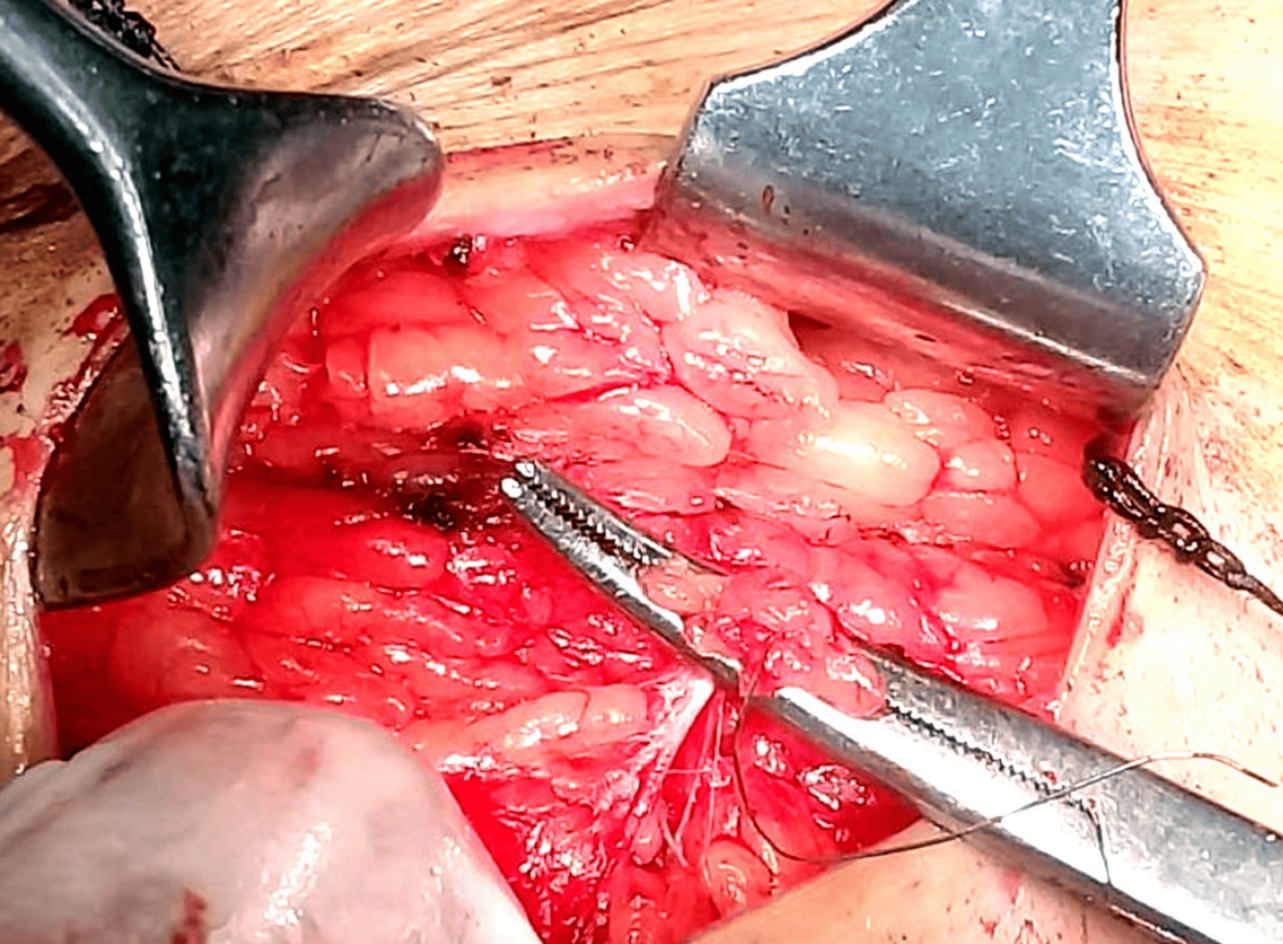
Complete delivery of the localization wire into the wound, marking the tract for excision

The wire was gradually delivered into the wound, as shown in Figures 8 and 9, allowing excision along its tract.

**Figure 10:**
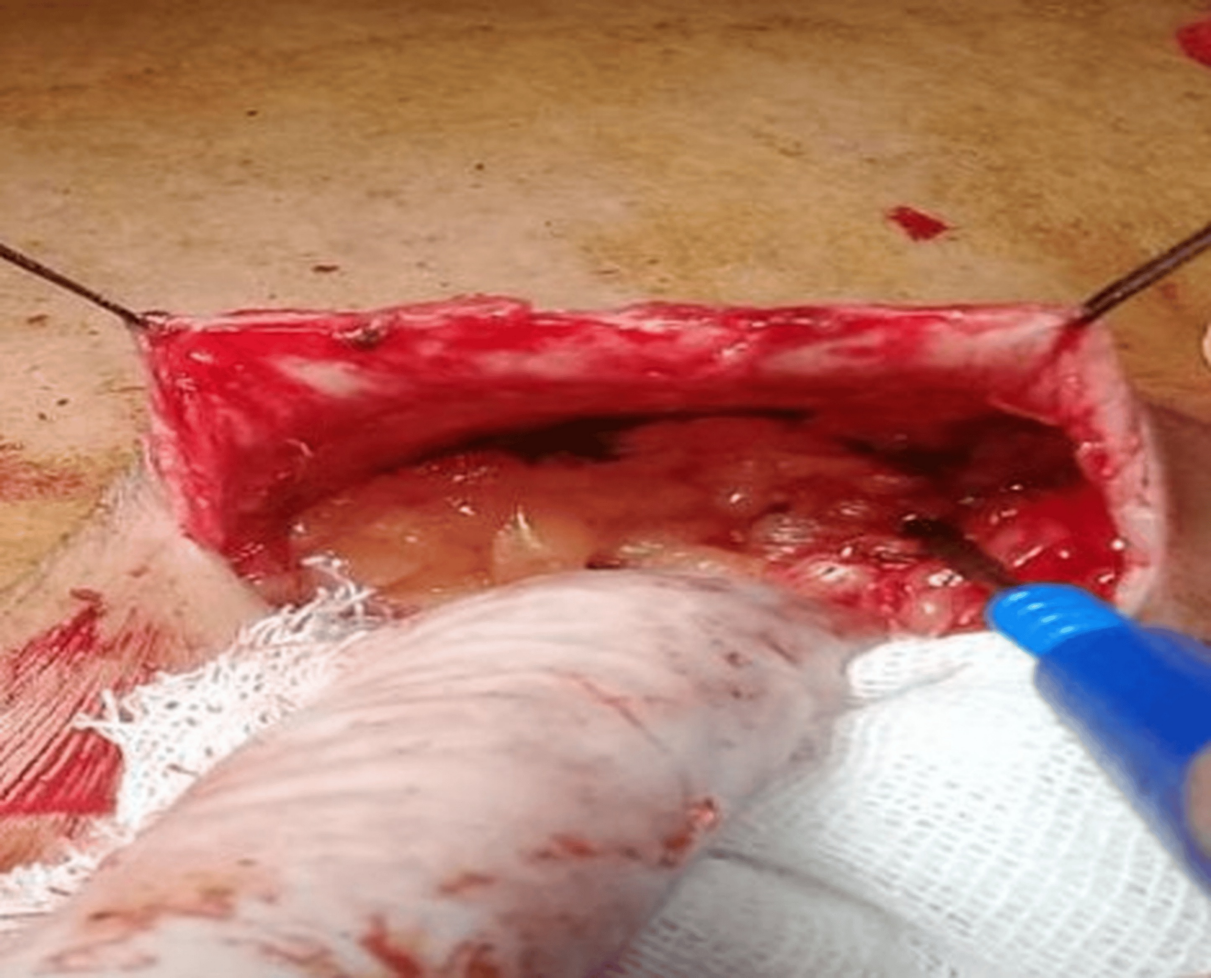
Circumferential injection of an aqueous carbon suspension around the lesion to mark its margins

Dissection of the breast parenchyma was carried out around the wire. taking care to not expose the wire and to ensure an adequate margin of healthy tissue around it. In some cases, an aqueous carbon suspension was injected circumferentially around the lesion to enhance visualization of the lesion boundaries and ensure clear margins, as shown in Figure 10.

**Figure 11:**
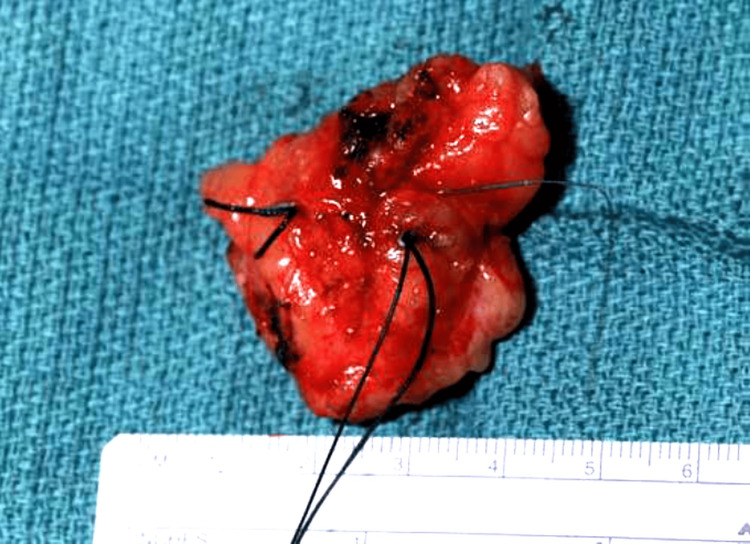
Excised specimen with the localization wire in situ, oriented with sutures to mark the anatomical margins

Dissection was continued down to the pectoral fascia. After the lesion and wire were completely excised, the specimen (with the wire still in situ) was oriented with anatomical markers for pathology, as shown in Figure 11.

**Figure 12:**
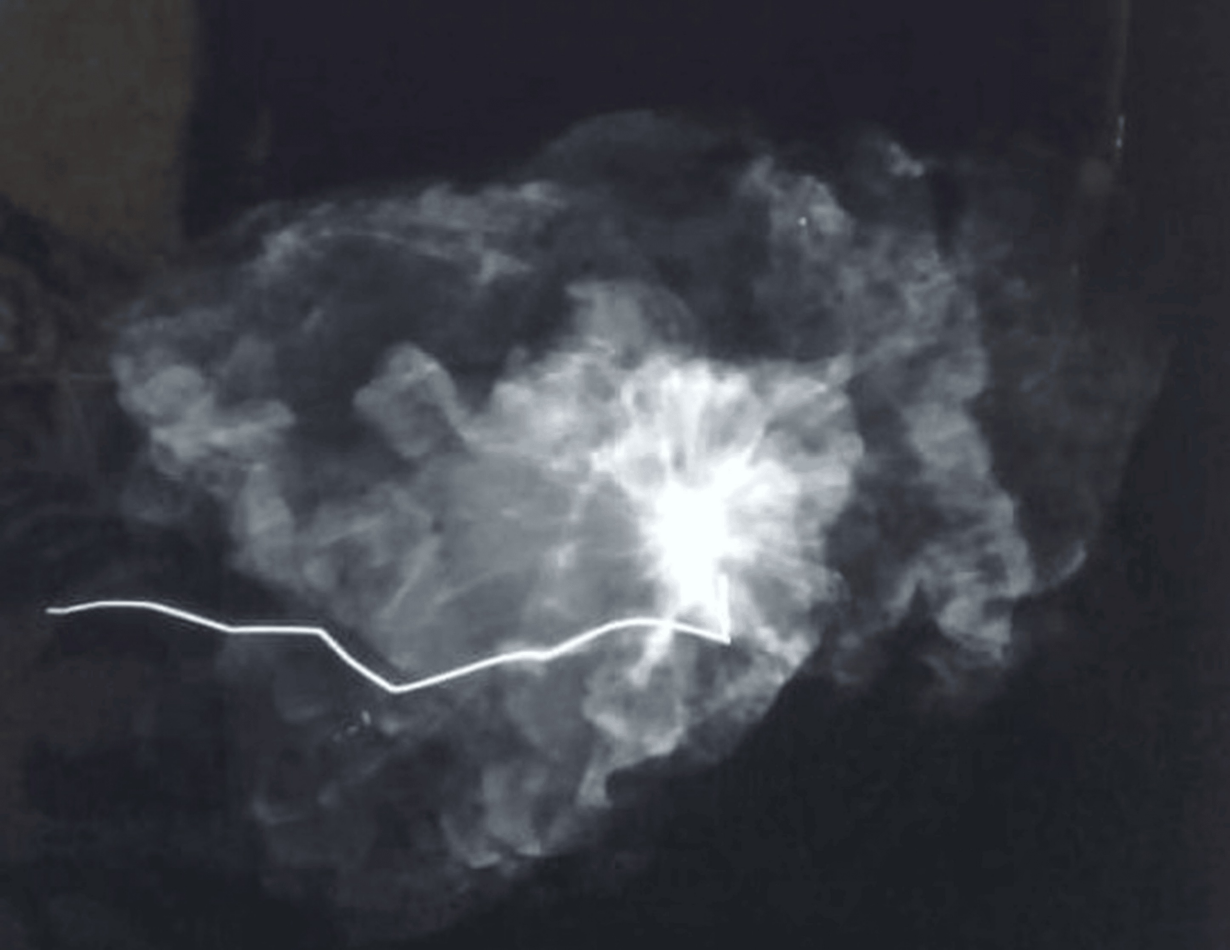
Specimen radiograph confirming removal of the entire target lesion and the localization wire

**Figure 13:**
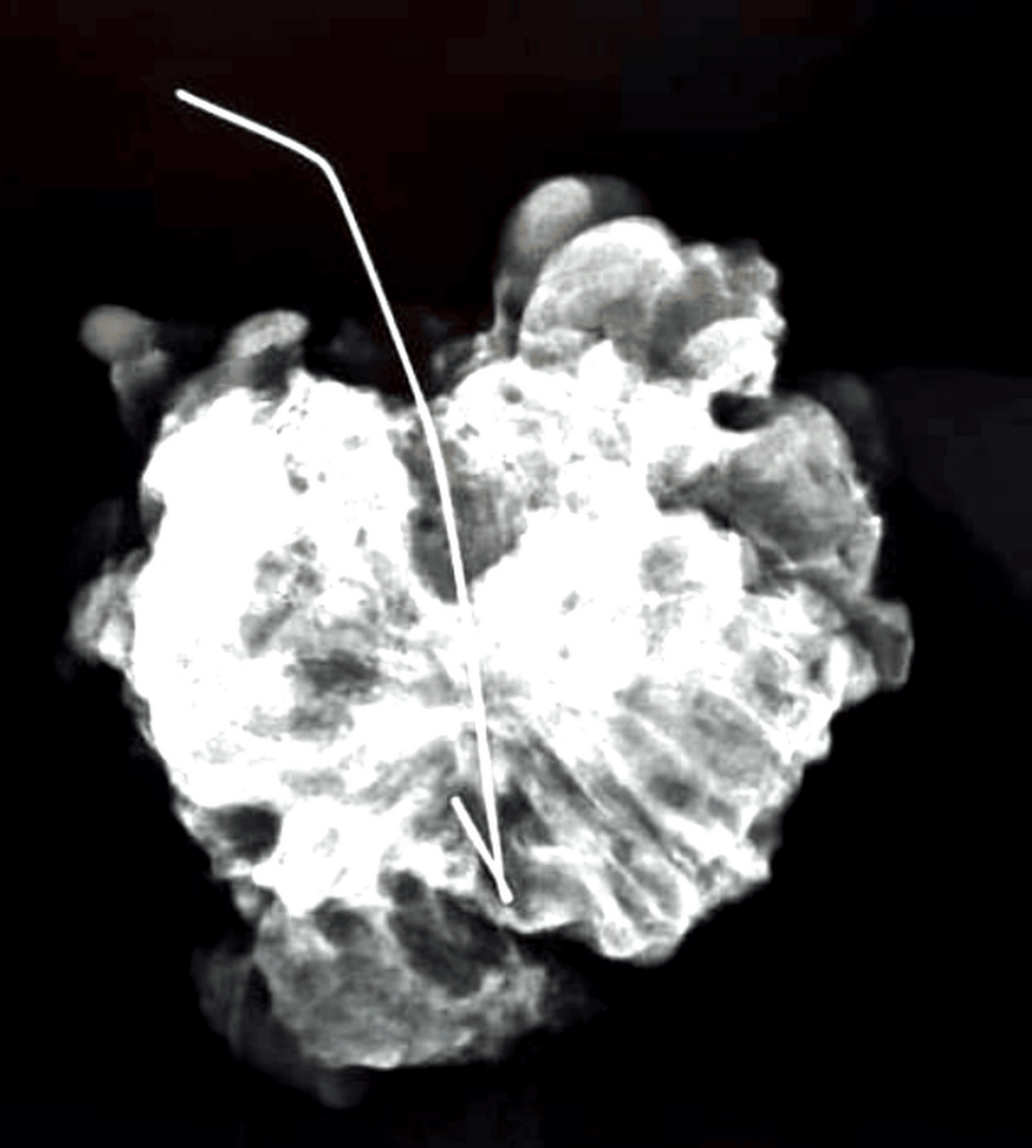
Second specimen radiograph verifying complete removal of the localization wire and the target lesion

Routine specimen radiography confirmed that the entire wire and lesion had been removed, as shown in Figures 12 and 13.

**Figure 14:**
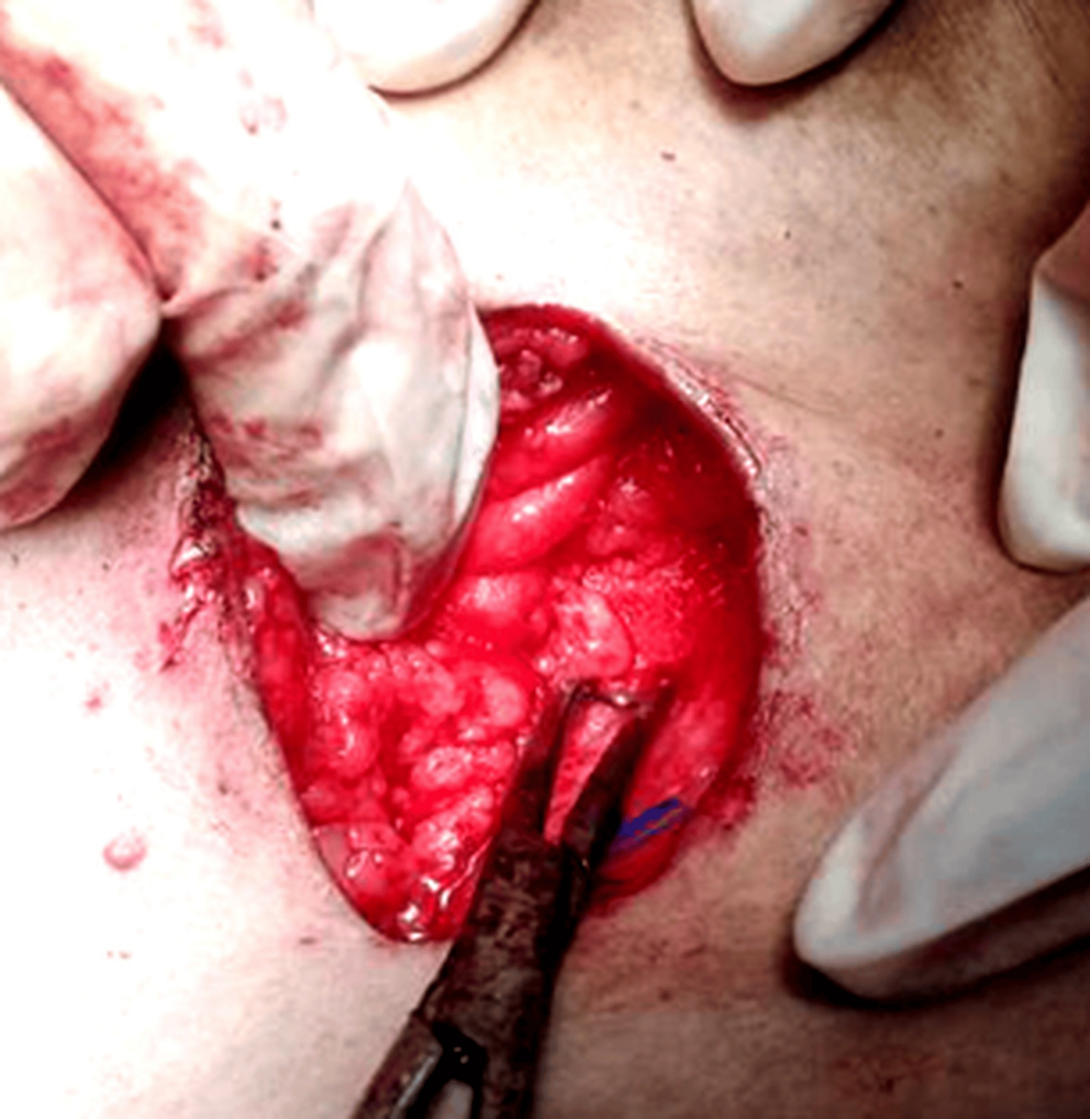
Placement of titanium clips in the lumpectomy cavity to mark the excision site for subsequent radiotherapy

Finally, titanium clips were inserted into the lumpectomy cavity to facilitate subsequent radiotherapy, as shown in Figure 14.

## Results

A total of 46 cases were included in this study, and all were female patients. Patient ages ranged from 34 to 75 years, with a mean age of 51.8 years (standard deviation ±9.8). All lesions were detected through screening imaging; none of the patients had any prior history of breast cancer or breast radiotherapy. Each patient underwent breast-conserving surgery (lumpectomy) with wire localization as described. Preoperative core needle biopsies confirmed malignancy in all cases (invasive carcinoma and/or ductal carcinoma in situ).

Localization technique

Most patients (36 of 46, 78.3%) underwent localization of a single lesion with a single wire. Four patients (8.7%) had two separate ipsilateral lesions, each localized with its own wire (two wires in one operation). Another four patients (8.7%) required bracketing wire localization, in which two wires were placed to bracket a single extensive area of non-palpable architectural distortion or segmental microcalcifications. In addition, in two cases (4.3%), a second wire had to be placed for a single lesion due to technical difficulties (e.g., the first wire placement was suboptimal and a second wire was added for accurate targeting). Table 1 summarizes the distribution of wire localization approaches in the cohort.

**Table 1:** Distribution of wire localization techniques (N=46)

Localization approach	Number of patients	Percentage of cohort
Single lesion localized with a single wire	36	78.30%
Two separate lesions (two wires in one patient)	4	8.70%
Bracketing wires for one extensive lesion	4	8.70%
Two wires for one lesion (due to placement difficulty)	2	4.30%

All wire localization procedures were successful. There were no instances of wire displacement or migration between the time of placement and surgery. In cases with bracketing, the multiple wires successfully outlined the lesion boundaries. In a subset of patients, an aqueous carbon suspension was injected around the lesion intraoperatively to assist in identifying the lesion. This technique was utilized in approximately nine cases (~20% of the cohort), particularly for lesions that were poorly visualized or had diffuse borders on imaging. In all 46 cases, the targeted lesions were successfully excised in the initial surgery. Specimen radiographs confirmed that the hooked wire (or wires) and the corresponding lesion were completely removed in every case (Figures 13, 14). The mean weight of the excised specimens was 34 g, with the weight of the specimens ranging from approximately 15 g for the smallest lesions to about 75 g for the largest excised area (wide local excisions for extensive microcalcifications).

Margin status and re-excision

Final histopathological analysis of the lumpectomy specimens showed clear (negative) margins in 43 out of 46 cases (93.5%) after the initial excision. In three cases (6.5%), the lumpectomy margins were positive for ductal carcinoma in situ (DCIS), meaning microscopic DCIS extended to one or more inked specimen margins. No invasive carcinoma was present at any margin in any case. All three patients with DCIS-positive margins underwent a re-excision lumpectomy to remove additional tissue at the affected margins. Each re-excision procedure confirmed residual DCIS confined to the area of the previous positive margin and achieved clear margins thereafter. No patient required more than one re-excision, and none required conversion to mastectomy.

All the three patients who required re-excision achieved clear margins in the second surgery, and thus all 46 patients ultimately obtained complete tumor removal with breast conservation. The overall re-excision rate in this series was 6.5% (3/46).

Postoperative outcomes

There were no significant intraoperative or postoperative complications in this series. In particular, no wires fractured or were lost during surgery, and no localized hematomas requiring re-operation occurred. Minor postoperative issues such as mild bruising or small seromas at the lumpectomy site were noted in a few patients. These were managed conservatively and did not delay adjuvant therapy. All patients proceeded to receive adjuvant treatment (radiotherapy and/or systemic therapy as indicated) without any delays attributable to the localization procedure or re-excisions. Cosmetic outcomes were not formally measured in this study, but qualitatively all patients achieved acceptable cosmetic results given the minimal volume of tissue removed (average specimen ~34 g) and the strategic incision planning used.

## Discussion

Mammographic screening programs have significantly increased the detection of impalpable invasive and in situ breast cancers, necessitating image-guided techniques to ensure precise surgical excision. Preoperative placement of a hook wire into non-palpable lesions under imaging guidance is a widely utilized method for patients with suspicious findings on mammography and/or ultrasound. This technique is particularly effective in localizing small lesions and microcalcifications, facilitating accurate surgical removal [1]. Despite its advantages, wire localization has certain drawbacks, including the potential for wire displacement and difficulty in repositioning, which may result in suboptimal outcomes, involved margins, and excessive excision of normal breast tissue [1]. Despite widespread WGL use, a majority of breast surgeons have expressed a preference to switch to alternative localization techniques [4]. According to Ahmed et al., re-operation rates for incomplete tumor excision with WGL have been reported in the literature to range between 10% and 43% for invasive and in-situ disease [5].

Case-control studies have compared ROLL with WGL. One such study reported a re-excision rate of 40% with WGL versus 16% with ROLL [6]. Another analysis found a re-excision rate of 21% with WGL versus 11% with ROLL [6]. In another report, the re-excision rate for WGL was 14.3% (two of 14 cases) compared to 10% (one of 10 cases) for ROLL [6]. Ocal et al. (2011) conducted a randomized trial comparing ROLL to WGL and found an involved margin rate of 8.3% in the WGL cases [7].

Several studies have evaluated alternative localization techniques. Krekel et al. (2011) compared IOUS with WGL in malignant lesions and found superior outcomes with IOUS; the re-excision rate was notably lower in the IOUS group (3.7%) compared to WGL (21.7%) [8]. Likewise, Arentz et al. (2010) reported a significantly higher re-excision rate with WGL (47%) compared to IOUS (24%) in the excision of non-palpable lesions (including benign and malignant cases) [9]. In contrast, a case-control study by Barentsz et al. (2012) observed a lower re-excision rate with wire-guided surgery (6.5%) compared to ultrasound-guided surgery (12.5%) [10]. Our case series showed a re-excision rate of 6.5%, aligning with the findings by Barentsz et al.

Kasem et al. (2020) analyzed 842 reflector insertions across 11 studies, reporting a 99.6% success rate for both the deployment and retrieval using the Savi Scout® (Merit Medical Systems, Inc., South Jordan, UT, US) radar localization. In a pooled analysis of four studies, Savi Scout was associated with significantly lower re-excision rates compared to WGL (12.9% vs. 21.1%, p<0.01) [1,4]. These results suggest that Savi Scout is a safe, effective alternative to WGL, offering greater scheduling flexibility and potentially reducing re-excision rates [11].

Early clinical evidence also supports the feasibility and reliability of wireless, non-radioactive localization systems for non-palpable breast lesions. A first-in-human study (Look Hong et al., 2020) evaluating the Magnetic Occult Lesion Localization Instrument (MOLLI) reported 100% successful excision and negative margins in all cases, with high user satisfaction among radiologists, surgeons, and pathology staff. These findings reinforce the growing evidence that wireless systems can offer safe, accurate, and user-friendly alternatives to WGL [12].

Tayeh et al. (2021) conducted a systematic review evaluating wireless localization technologies, including the radiofrequency identification (RFID) device LOCalizer™ (Hologic Inc., Santa Clara, CA, US). Across seven studies (1,151 patients, 1,344 RFID tags), the pooled deployment success was 99.1% with a retrieval rate of 100%. The overall re-excision rate was 13.9%, with only one reported complication. Two studies directly compared RFID with WGL, finding identical re-excision rates of 15.6% (p=0.995), indicating no significant difference in efficacy. The authors concluded that the LOCalizer™ was a safe and non-inferior alternative to WGL. However, further research is needed to assess cost-effectiveness and cosmetic outcomes relative to WGL and other wireless technologies [13].

The UK implant Breast Reconstruction evaluation-NETwork (iBRA-NET) localization study demonstrated safety and effectiveness comparable to wire localization, with a re-excision rate of ~12% for both techniques. This study established a robust platform for the comparative evaluation of new localization devices [14].

A systematic review and meta-analysis of IOUS in breast cancer surgery showed that IOUS is associated with significantly lower positive margin and re-excision rates compared to WGL. Three randomized controlled trials reported higher negative margin rates with IOUS, and a pooled analysis confirmed its superiority (risk ratio 4.34, p<0.0001). Additionally, evidence from 41 cohort studies (3,291 patients) supported improved surgical outcomes with IOUS [15].

In a 2012 randomized multicenter trial comparing ROLL with WGL in breast-conserving surgery, re-excision was required in 12% of patients in the ROLL group and 10% in the WGL group, with no statistically significant difference between the two techniques [16]. Similarly, a prospective randomized trial comparing RSL with WGL found no significant difference in positive margin rates (10.5% vs 11.8%, respectively), overall reoperation rates, specimen size, or postoperative complications [17].

We attribute the low re-excision rate in our study (6.5%) to effective collaboration between radiologists and surgeons, facilitated by clear communication and standardized terminology for describing the wire’s trajectory relative to the lesion. Radiologists employed a clock-face reference system, designating the wire entry site as the center and specifying its length within the breast, as well as the distance between the entry point and the corresponding skin location of the lesion. This standardized approach ensured consistency in surgical technique and facilitated reproducibility. Whenever feasible, the incision was strategically planned based on the skin mark corresponding to the lesion. Routine specimen radiography was performed to confirm the complete removal of both the hook wire and the lesion. Additionally, shave margins were taken with intraoperative frozen section analysis to enhance surgical precision. Adherence to this protocol contributed to the lower re-excision rate observed in our study.

## Conclusions

Wire localization is a reliable technique for excising non-palpable, screen-detected breast cancers. Its precision is enhanced by a comprehensive preoperative diagnostic workup, including tru-cut biopsy and appropriate imaging studies, and by close collaboration between surgeons and radiologists to accurately delineate the lesion’s extent and guide optimal wire placement. Careful incision planning, wide excision margins, and intraoperative frozen section analysis all contribute to reduced re-excision rates. The use of multiple wires to bracket extensive lesions can further improve margin clearance. Overall, although wire localization is technically demanding and requires meticulous preoperative assessment and skilled execution, it offers reproducible results when coupled with specimen imaging and intraoperative pathology evaluation.
